# Donor heart preservation with hypoxic-conditioned medium-derived from bone marrow mesenchymal stem cells improves cardiac function in a heart transplantation model

**DOI:** 10.1186/s13287-020-02114-7

**Published:** 2021-01-13

**Authors:** Pengyu Zhou, Hao Liu, Ximao Liu, Xiao Ling, Zezhou Xiao, Peng Zhu, Yufeng Zhu, Jun Lu, Shaoyi Zheng

**Affiliations:** 1 Department of Cardiovascular Surgery, Nanfang Hospital, Southern Medical University, No.1838 North Guangzhou Avenue, Baiyun District, Guangzhou, People’s Republic of China; 2 Laboratory Animal Research Center, Nanfang Hospital, Southern Medical University, No.1838 North Guangzhou Avenue, Baiyun District, Guangzhou, People’s Republic of China

**Keywords:** Mesenchymal stem cells, Hypoxic preconditioning, Conditioned medium, Secretome, Ischemia/reperfusion injury, Heterotopic heart transplantation

## Abstract

**Background:**

In heart transplantation, donor hearts inevitably suffer from ischemia/reperfusion (I/R) injury, which leads to primary graft dysfunction and affects patients’ survival rate. Bone marrow mesenchymal stem cells (BMSCs) have been reported to attenuate myocardial I/R injury via their paracrine effects, which can be enhanced by hypoxic preconditioning. We hypothesized that the donor heart preservation with hypoxic conditioned medium (CdM) derived from BMSCs would improve post-transplant graft function.

**Methods:**

Normoxic or hypoxic CdM were isolated from rat BMSCs cultured under normoxic (20% O_2_) or hypoxic (1% O_2_) condition. Donor hearts were explanted; stored in cardioplegic solution supplemented with either a medium (vehicle), normoxic CdM (N-CdM), or hypoxic CdM (H-CdM); and then heterotopically transplanted. Antibody arrays were performed to compare the differences between hypoxic and normoxic CdM.

**Results:**

After heart transplantation, the donor heart preservation with normoxic CdM was associated with a shorter time to return of spontaneous contraction and left ventricular systolic diameter, lower histopathological scores, higher ejection fraction, and fractional shortening of the transplanted hearts. The cardioprotective effects may be associated with the inhibition of apoptosis and inflammation, as reflected by less TUNEL-positive cells and lower levels of plasma proinflammatory cytokines (interleukin-1β, interleukin-6, tumor necrosis factor-α) and cardiac troponin I in the N-CdM group compared with the vehicle group. These therapeutic effects can be further enhanced by hypoxic preconditioning. Antibody arrays revealed that nine proteins were significantly increased in hypoxic CdM compared with normoxic CdM. Furthermore, compared with vehicle and N-CdM groups, the protein levels of PI3K and p-Akt/Akt ratio in the transplanted hearts significantly increased in the H-CdM group. However, no significant difference was found in the phosphorylation of Smad2 and Smad3 for the donor hearts among the three groups.

**Conclusions:**

Our results indicate that the cardioplegic solution-enriched with hypoxic CdM can be a novel and promising preservation solution for donor hearts.

## Introduction

Heart transplantation remains a well-established therapy of choice for patients with refractory heart failure [1]. Despite the increasing population of heart failure patients, the number of heart transplantation has not increased proportionally over the past 2 decades due to the shortage of donor hearts [2]. Additionally, 5 to 10% of recipients have suffered from primary graft dysfunction (PGD) after heart transplantation [3].

Previous studies have shown that ischemia/reperfusion (I/R) injury plays a significant role in PGD [4], thereby contributing to adverse short- and long-term clinical outcomes in the recipients. The key mechanisms underlying myocardial I/R injury include increased intracellular calcium concentration, sudden generation of reactive oxygen species (ROS) and inflammatory cytokines, adenosine triphosphate (ATP) depletion, and development of metabolic acidosis. All of these factors are known to result in myocardial apoptosis [5] and the acceleration of allograft rejection or chronic allograft dysfunction. Therefore, attenuating myocardial I/R injury during the heart transplant procedure would have a favorable impact on improving short- and long-term graft function and recipient’s survival [6].

The administration of bone marrow-derived mesenchymal stem cells (BMSCs) is emerging as a promising approach for I/R injury-induced myocardial infarction due to its multiple differentiation potential [7, 8]. However, recent reports have demonstrated paracrine activities are likely implicated in the therapeutic effects of BMSCs [9] due to the relatively low long-term survival rate of implanted BMSCs [10, 11]. Increasing evidence has shown that BMSCs secrete a vast array of bioactive factors, such as chemokines/cytokines [12], anti-apoptotic, and growth factors [13, 14]. In line with the paracrine hypothesis, hypothermic perfusion of donor hearts with conditioned medium (CdM) from BMSCs (CdM-BMSCs) could protect against myocardial I/R injury in old rats (15-month old) [15]. Additionally, the preservation of brain-dead donor hearts with cardioplegic solution-supplemented with CdM-BMSCs improved graft contractility after transplantation [16]. Notably, recent studies demonstrated that hypoxic preconditioning BMSCs intensified their paracrine abilities by secreting more nutritional bioactive factors, such as vascular endothelial growth factor (VEGF), hepatocyte growth factor (HGF), and Activin A [17, 18]. It has been shown that the administration of hypoxic CdM-BMSCs can reduce tissue damage and improve neurological recovery after ischemic stroke [19]. Roth et al. reported that the injection of hypoxic CdM-BMSCs significantly restored retinal function and attenuated apoptosis after retinal ischemia [20]. Additionally, hypoxic CdM-BMSCs have been found to alleviate oxygen-glucose deprivation/reoxygenation-induced injury and promote the anti-inflammatory polarization of microglia due to the beneficial effect of exosome [21]. However, no studies have applied hypoxic CdM-BMSCs into Custodiol cardioplegic solution as a preservation solution for cold storage of donor hearts.

Therefore, in the current study, we hypothesized that CdM-BMSCs under normoxic conditions (20% O_2_ BMSCs) added to a preservation solution could improve in vivo left ventricular (LV) graft dysfunction after heart transplantation and that these cardioprotective effects could be enhanced by hypoxic preconditioning (1% O_2_ BMSCs) in the well-established heterotopic heart transplantation model [22–24].

## Materials and methods

### Animals

Inbred male Lewis rats (8–12 weeks old; Charles River Laboratories, Beijing, China) received care in compliance with the Guide for the Care and Use of Laboratory Animals (National Institutes of Health Publication No. 85-23, revised 1996). All procedures and handling of animals during the investigations were reviewed and approved by the Ethical Committee of Laboratory Animal Research Center of Southern Medical University Nanfang Hospital. The animals were housed at constant ambient temperature (22 ± 2 °C) in light-controlled rooms (12–12-h light-dark cycles), were given food and water access ad libitum, and acclimatized for 1 week.

### Experimental groups

Rats were randomly assigned to three groups. The donor hearts were arrested and stored for 1 h in either cold Custodiol (Dr. Franz Köhler, Chemie GmbH, Bensheim, Germany) supplemented with a serum-free medium vehicle (α-MEM) (vehicle group, 8 rats), or Custodiol supplemented with normoxic CdM-BMSCs (N-CdM group, 8 rats), or Custodiol supplemented with hypoxic CdM-BMSCs (H-CdM group, 8 rats). Then, the donor hearts were heterotopically transplanted.

### Isolation and culture of BMSCs

As previously reported [15], BMSCs were harvested from the bone marrow of 8-week-old male Lewis rats. Briefly, rats were euthanized with an overdose of pentobarbital sodium (100 mg/kg, intraperitoneally). The bone marrow was isolated by flushing femurs and tibias with phosphate-buffered saline (PBS, Life Technologies, Grand island, NY, USA). The cells were suspended in MEM Alpha basic (1X) (α-MEM, Life Technologies, Grand island, NY, USA) supplemented with 10% fetal bovine serum (Life Technologies, Grand island, NY, USA), 1% penicillin-streptomycin (Life Technologies, Grand island, NY, USA), and then incubated at 37 °C with 5% CO_2_ on culture flasks. The primary culture was subcultured at a ratio of 1:2 when 80% confluency was reached. Only the third passage was used in subsequent experiments.

### Preparation of hypoxic and normoxic CdM-BMSCs

**Fig. 1:**
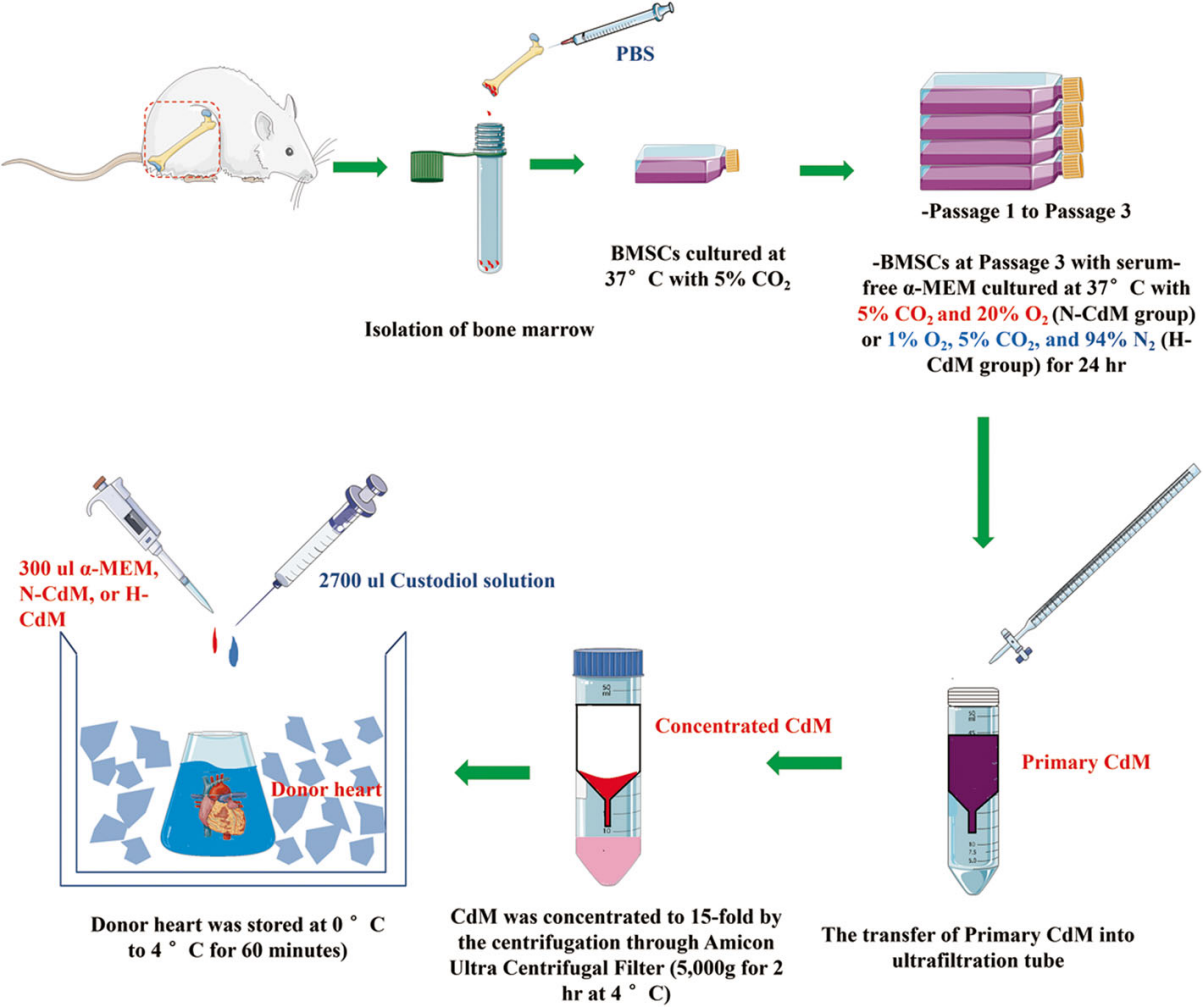
Schematic shows an overview of steps for the collection of normoxic or hypoxic CdM-BMSCs. After BMSCs reached greater than 80% confluency at Passage 3, the medium was aspirated, and BMSCs were rinsed 3 times with PBS. Then, α-MEM was added to culture flasks with BMSCs, and the culture flasks were put into an incubator under the normoxic or hypoxic conditions for 24 h. For the normoxic group, BMSCs were cultured at 37 °C in a humidified atmosphere containing 5% CO_2_ and 20% O_2_. The hypoxic group was cultured at 37 °C in 1% O_2_, 5% CO_2_, and 94% N_2_ in a hypoxic incubator. The primary CdM was collected after incubation for 24 h, and the cell debris was removed using a syringe filter. The primary CdM was then sequentially concentrated to 15-fold by the centrifugation through Amicon Ultra Centrifugal Filter (5000 g for 2 h at 4 °C) according to the manufacturer’s protocol. Finally, the novel donor heart preservation solution was produced by diluting 300 ul α-MEM (Vehicle group), concentrated normoxic (N-CdM group), or hypoxic CdM (H-CdM group) from the filtrate tube of the top unit in 2700-ul Custodiol cardioplegic solution. BMSCs bone marrow mesenchymal stem cells, CdM conditioned medium, CdM-BMSCs conditioned medium-derived from bone marrow mesenchymal stem cells, N-CdM normoxic conditioned medium, H-CdM hypoxic-conditioned medium

Normoxic and hypoxic CdM-BMSCs were acquired as described previously with slight modifications [19]. A simplified schematic of the CdM-BMSCs collection protocol is shown in Fig. 1. After BMSCs reached greater than 80% confluency at passage 3, the medium was aspirated, and BMSCs were rinsed 3 times with PBS. Then, α-MEM was added to culture flasks with BMSCs, and the culture flasks were put into an incubator under the normoxic or hypoxic conditions for 24 h. For the normoxic group, BMSCs were cultured at 37 °C in a humidified atmosphere containing 5% CO_2_ and 20% O_2_. The hypoxic group was cultured at 37 °C in 1% O_2_, 5% CO_2_, and 94% N_2_ in a hypoxic incubator (Galaxy 48, Eppendorf, Shanghai, China). The primary CdM was collected after incubation for 24 h, and the cell debris was removed using a syringe filter (Millex-GP; Millipore, Burlington, MA, USA). The primary CdM-BMSCs was then sequentially concentrated to 15-fold by the centrifugation through Amicon Ultra Centrifugal Filter (5000 g for 2 h at 4 °C, Amicon Ultra-15 3 K, Millipore, Burlington, MA, USA) according to the manufacturer’s protocol. Finally, the novel donor heart preservation solution was produced by diluting 300 ul α-MEM (vehicle group), concentrated normoxic (N-CdM group), or hypoxic CdM-BMSCs (H-CdM group) from the filtrate tube of the top unit in 2700-ul Custodiol cardioplegic solution as indicated in Fig. 1. α-MEM was regarded as a control (nonconditioned medium).

### Screening of secreted proteins in CdM-BMSCs

The cytokines/chemokines of isolated CdM-BMSCs were measured by using a G-series rat cytokine array 67 (Raybiotech, Norcross, GA, USA) according to the manufacturer’s instructions. The Gene Ontology (GO) analysis (R package “org.Hs.eg.db” and “clusterProfiler”) and Kyoto Encyclopedia of Genes and Genomes (KEGG) pathway enrichment analysis (R package “clusterProfiler”) were performed using the open-source program R (version 3.5.1) to investigate the biological process categories and pathways of proteins that were highly expressed between hypoxic and normoxic CdM-BMSCs, as previously described [25].

### The rat model of heterotopic heart transplantation

#### Surgical technique of heart transplantation

As described elsewhere [24, 26], heterotopic heart transplantations were performed in an isogenic Lewis-to-Lewis rat strain model to exclude the effects of immune rejections and avoid the application of immunosuppressors such as cyclosporine.

**Fig. 2:**
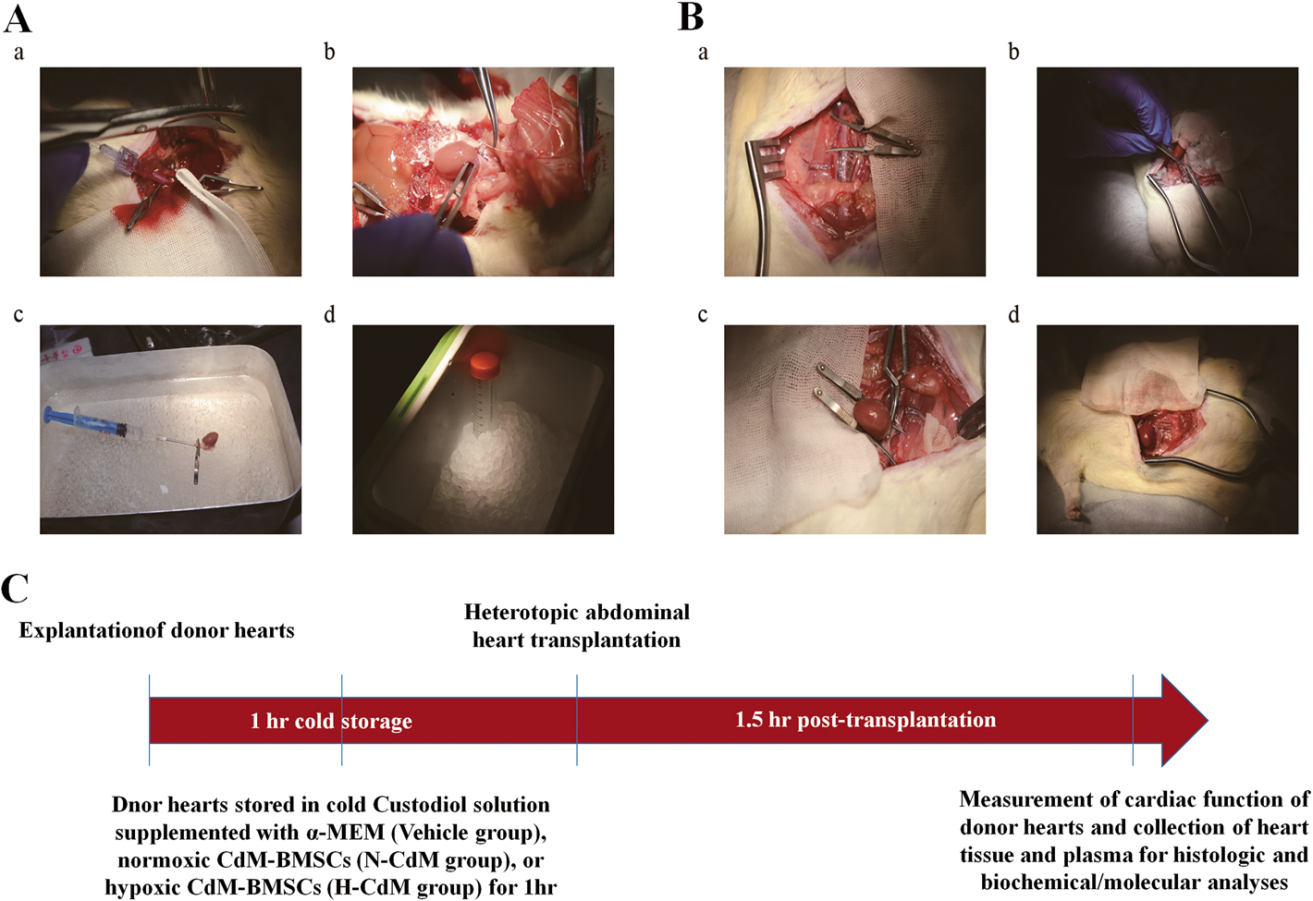
The representative and important steps of the donor heart explantation, transplantation and the experimental protocol. **a** a: Abdominal artery cannulation was performed. b: The donor heart was explanted. c: 1 ml cold Custodiol solution supplemented with α-MEM or CdM-BMSCs was applied to perfuse slowly and retrogradely the donor heart via the aorta. d: The donor heart was stored in the cold Custodiol solution supplemented with α-MEM or CdM-BMSCs at 0 °C to 4 °C for 60 min. **b** a: 1.5 cm abdominal artery and inferior vena cava below the level of the renal artery were carefully clamped by two vessel clamps. **b**: End-to-side anastomoses were performed. c: End-to-side anastomoses were completed. d: The donor heart was in-vivo reperfused. **c** The donor heart was explanted, stored in cold Custodiol solution supplemented with α-MEM or CdM-BMSCs for 1 h, and transplanted. After 1.5 h post-transplantation, in vivo left-ventricular graft function was evaluated. Myocardial samples and plasma were collected for biochemical analysis. CdM-BMSCs: conditioned medium derived from bone marrow mesenchymal stem cell; N-CdM, normoxic conditioned medium; H-CdM, hypoxic conditioned medium

As shown in Fig. 2a, heart explantation was performed as follows. Briefly, donor rats were anesthetized with pentobarbital sodium (60 mg/kg, intraperitoneally) and only allowed to be operated on when no palpebral relex of donor rats was observed. After that, both the abdominal artery and inferior vena cava were exposed by careful dissection with the cotton swab after the abdominal cavity was opened. A 0.6-ml saline with a high dose of heparin (6250 IU/kg) was slowly injected via inferior vena cava. Abdominal artery cannulation was performed after heparinization. The chest cavity of the donor rat was open, and the inferior vena cava was cut. Then, the donor heart was immersed completely with ice and cold Custodiol cardioplegic solution was slowly and retrogradely perfused into the donor heart via the abdominal artery within 3 min. After that, inferior and superior vena cava, pulmonary veins were ligated and cut. Aorta and pulmonary artery were dissected and cut, respectively. Finally, the donor heart was taken out from the chest cavity. A 1-ml cold Custodiol solution supplemented with α-MEM or CdM-BMSCs was applied to perfuse slowly and retrogradely the donor heart via the aorta. After that, the donor heart was statically stored in the Custodiol solution supplemented with α-MEM or CdM-BMSCs (2700-ul Custodiol solution with 300 ul α-MEM or CdM-BMSCs) at 0 to 4 °C for 60 min.

The degree of rat cardiac allograft injury correlates with increased cold ischemia time [27]; therefore, to minimize the variability among experiments, the duration between harvest, cold storage, and reperfusion was standardized to 110 min (10 min (harvest of the donor hearts) + 60 min (cold storage of the donor hearts) + 40 min (heart transplantation)) as previously described [28].

As shown in Fig. 2b, heterotopic abdominal heart transplantation was performed as follows. Recipient rats were anesthetized with isoflurane (5% for induction and 2% for maintenance of anesthesia). Firstly, left external jugular vein cannulation was performed in the recipient rat so that 1-ml saline with a low dose of heparin (250 IU/kg) could be administered. Both the abdominal artery and inferior vena cava were exposed by careful dissection with the cotton swab after the abdominal cavity was opened. A 1.5-cm abdominal artery and inferior vena cava below the level of the renal artery were carefully clamped by two delicate vessel clamps. All the branches of both the abdominal artery and inferior vena cava were tightly ligated. At the end of a 60-min cold storage of the donor hearts, 3 mm incisions were made in the abdominal artery and inferior vena cava. Then, the donor heart was wrapped by the moist gauze with cold Custodiol solution supplemented with α-MEM or CdM-BMSCs and placed next to the abdominal artery. Finally, end-to-side anastomoses were performed between the aorta and the abdominal aorta and between the pulmonary artery and the inferior vena cava with 8–0 prolene suture under the microscope. The vessel clamps were carefully released to reperfuse the donor heart after the completion of anastomoses. In vivo reperfusion lasted for 1.5 h after the donor heart was heterotopically transplanted into the recipient rat. The time to return of spontaneous contraction of the donor hearts immediately after successful heart transplantation was also recorded. The measurement of LV graft function and sample collection for histologic and molecular analyses were performed after 1.5 h reperfusion. The experimental protocol is shown in Fig. 2c.

#### Functional measurement in the graft

As previously reported [29], at 1.5 h after transplantation, transabdominal echocardiographic imaging was performed by a blinded observer using a dedicated Vevo® 2100 System (21 MHz broadband sector transducer, VisualSonics Inc., Toronto, Canada). Ejection fraction (EF), left ventricular internal diameter at end-diastole (LVIDd), and at end-systole (LVIDs), stroke volume (SV), heart rate (HR), and cardiac output (CO) were measured between the anterior wall and the posterior wall of the left ventricle from the short-axis view at the level of the papillary muscles from M-mode recordings. For each measurement, three consecutive cardiac cycles were traced and averaged. Fractional shortening (FS) was calculated as [(LVIDd-LVIDs)/LVIDd] × 100 (%).

#### Histology and immunohistochemistry in the graft

The donor hearts were explanted after the functional measurements. Pieces of myocardial tissue were fixed immediately in paraformaldehyde solution (4%) and embedded in paraffin. Five-micron-thick slices of myocardium were stained with hematoxylin and eosin. The pathology changes of each heart slice were evaluated by scoring according to the grades 0–4 [30]: (0) Nil, (1) minimum (focal myocyte damage), (2) mild (occasionally disordered myocardial fibers with multifocal myofibrillar degeneration and inflammatory process), (3) moderate (diffuse inflammation and/or comprehensive myofibrillar degeneration with wave-shaped myocardial fibers and shed nuclei), and (4) severe (diffuse inflammatory process with myocardial necrosis: the nuclei shrink and the cells are severely damaged). We selected four random and nonoverlapping visual fields for each heart slice under a light microscope, and the average histopathological score of four different fields was calculated for each sample in a blinded way.

#### Detection of DNA strand breaks in the graft

Terminal deoxynucleotidyl transferase dUTP nick end labeling (TUNEL) staining to detect DNA-strand breaks of the donor hearts after functional measurements was performed as previously described [23]. The number of TUNEL-positive cells was counted under a fluorescence microscope, and the final results were expressed as the ratio of 4′,6-diamidino-2-phenylindole (DAPI)-TUNEL double-labeled nuclei to the total number of nuclei stained with DAPI.

#### Proinflammatory cytokines and markers of myocardial injury

Blood samples from the recipient rats were obtained at 1.5 h after reperfusion just before the sacrifice of rats. Blood samples were drawn from the abdominal aorta. Then, plasma samples were obtained after centrifugation (3000 rpm, 15 min, 4 °C). The levels of proinflammatory cytokines (tumor necrosis factor (TNF)-α, interleukin (IL)-1β, IL-6) and cardiac troponin I (cTnI) were measured via Rat ELISA kit (R&D Systems, Inc., Minneapolis, MN, USA).

#### Western blotting

Myocardial protein expression was assessed by Western blot as previously described [16]. The ratio of phosphorylated-Akt to Akt, phosphorylated-Smad2 to Smad2, phosphorylated-Smad3 to Smad3, and phosphatidylinositol 3-kinase (PI3K) (1:1000 dilution, Cell Signaling Technology (Shanghai) Biological Reagents Company Limited, Shanghai, China) were calculated.

### Statistical analysis

The results were expressed as mean ± standard error of the mean (SEM). GraphPad Prism 7.02 software (GraphPad Sofware, Inc., San Diego, CA, USA) was used to perform statistical analysis. Shapiro-Wilk test was performed to test the normality of data before statistical tests were applied. For data with normal distribution, a two-sample Student’s *t* test was applied to analyze the difference between N-CdM and H-CdM groups. If the normality test failed, a nonparametric Mann-Whitney test was used. One-way ANOVA followed by Tukey’s post hoc test was performed for multiple comparisons between three experimental groups. If the data failed the normality test, the nonparametric Kruskal-Wallis test followed by Dunn’s post hoc test was used. A value of *p* < 0.05 was considered statistically significant.

## Results

### Effect of normoxic and hypoxic CdM-BMSCs on the transplanted heart

#### Time to return of spontaneous contraction

**Fig. 3:**
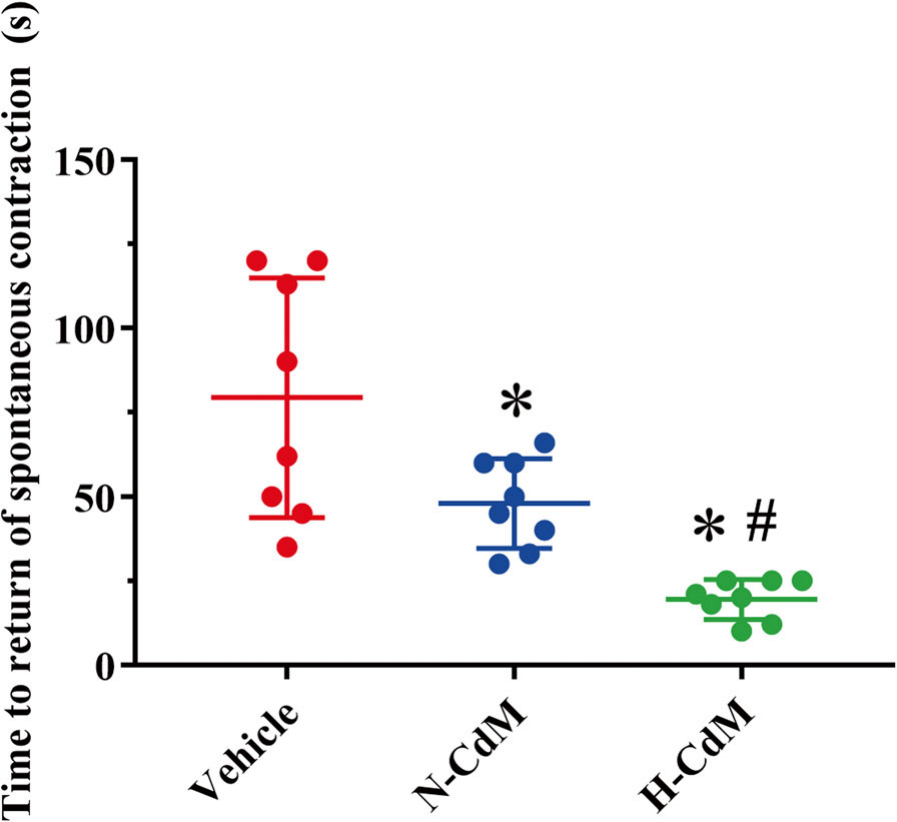
Effects of N-CdM and H-CdM on time to return of spontaneous contraction of the transplanted hearts after heart transplantation. N-CdM and H-CdM indicate normoxic- and hypoxic-conditioned medium, respectively. Data represent mean ± standard error of the mean. One-way ANOVA/Kruskal-Wallis was applied in statistical analysis. *p < 0.05 vs. vehicle, #*p* < 0.05 vs. N-CdM

Time to return of spontaneous contraction of the donor hearts immediately after successful heart transplantation was significantly decreased in the N-CdM and H-CdM groups compared to the vehicle group (Fig. 3). Besides, hypoxic CdM-BMSCs were associated with a significant shortening of time to return of spontaneous contraction of the grafts compared to the N-CdM group (Fig. 3).

#### LV graft function

**Fig. 4:**
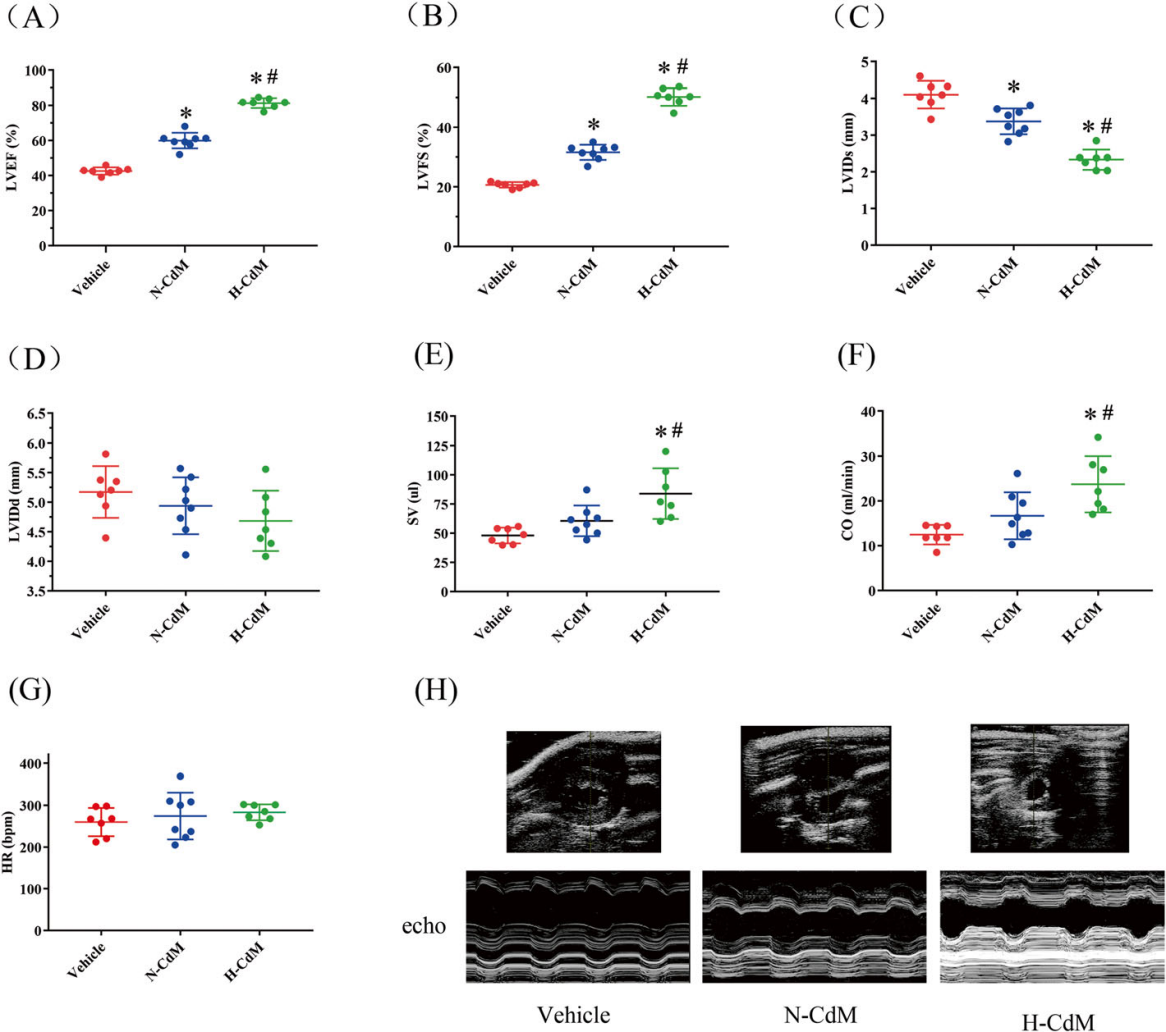
Changes in echocardiographic parameters in the transplanted hearts after cold storage with a preservation solution supplemented by N-CdM and H-CdM. The representative echocardiographic pictures are taken from M-mode recordings. Cardiac function and left ventricular size, as measured by echocardiographic parameters: **a** left ventricular ejection fraction (LVEF; as a percentage), **b** left ventricular fractional shortening (LVFS; as a percentage), **c** left ventricular internal dimension at end-systole (LVIDs; in millimeters), **d** left ventricular internal dimension at end-diastole (LVIDd; in millimeters), **e** stroke volume (SV; in microliter), **f** heart rate (HR; in beat per minute), **g** cardiac output (CO; in milliliter per minute), and **h** representative echocardiographic images of the transplanted heart in three groups. N-CdM and H-CdM indicate normoxic- and hypoxic-conditioned medium, respectively. Data represent mean ± standard error of the mean. One-way ANOVA/Kruskal-Wallis was applied in statistical analysis. *p < 0.05 vs. vehicle, #*p* < 0.05 vs. N-CdM

One transplanted graft in the vehicle and H-CdM group could not be included in the heart function analysis since part of the intestine covered the transplanted heart, making the evaluation of heart function by echocardiography very difficult and challenging. After heart transplantation, significantly increased left ventricular EF and FS were observed in N-CdM and H-CdM groups compared to the vehicle group (Fig. 4a, b). Moreover, the preservation of grafts with Custodiol solution supplemented with hypoxic CdM-BMSCs was associated with significantly increased left ventricular EF and FS in the donor hearts compared with normoxic CdM-BMSCs (Fig. 4a, b).

Significant improved myocardial recovery in N-CdM and H-CdM groups compared with the vehicle group was noted with regards to the increased LVIDs (Fig. 4c). Furthermore, hypoxic CdM-BMSCs exerted more robust effects than normoxic CdM-BMSCs with respect to the increased LVIDs (Fig. 4c). However, there was no significant difference in the LVIDd among the three groups (Fig. 4d).

Moreover, significantly increased SV and CO were observed in the H-CdM group compared to N-CdM and vehicle groups (Fig. 4e, f). While no significant difference was observed in the HR among the three groups (Fig. 4g).

In general, the preservation of the donor hearts with Custodiol solution supplemented with normoxic CdM-BMSCs appeared to protect cardiac contractility in the donor hearts subjected to acute I/R injury, and hypoxic CdM-BMSCs added in the preservation solution could further enhance this cardioprotective effect for grafts (Fig. 4h).

#### Histological and immunohistochemical evaluation of the graft

**Fig. 5:**
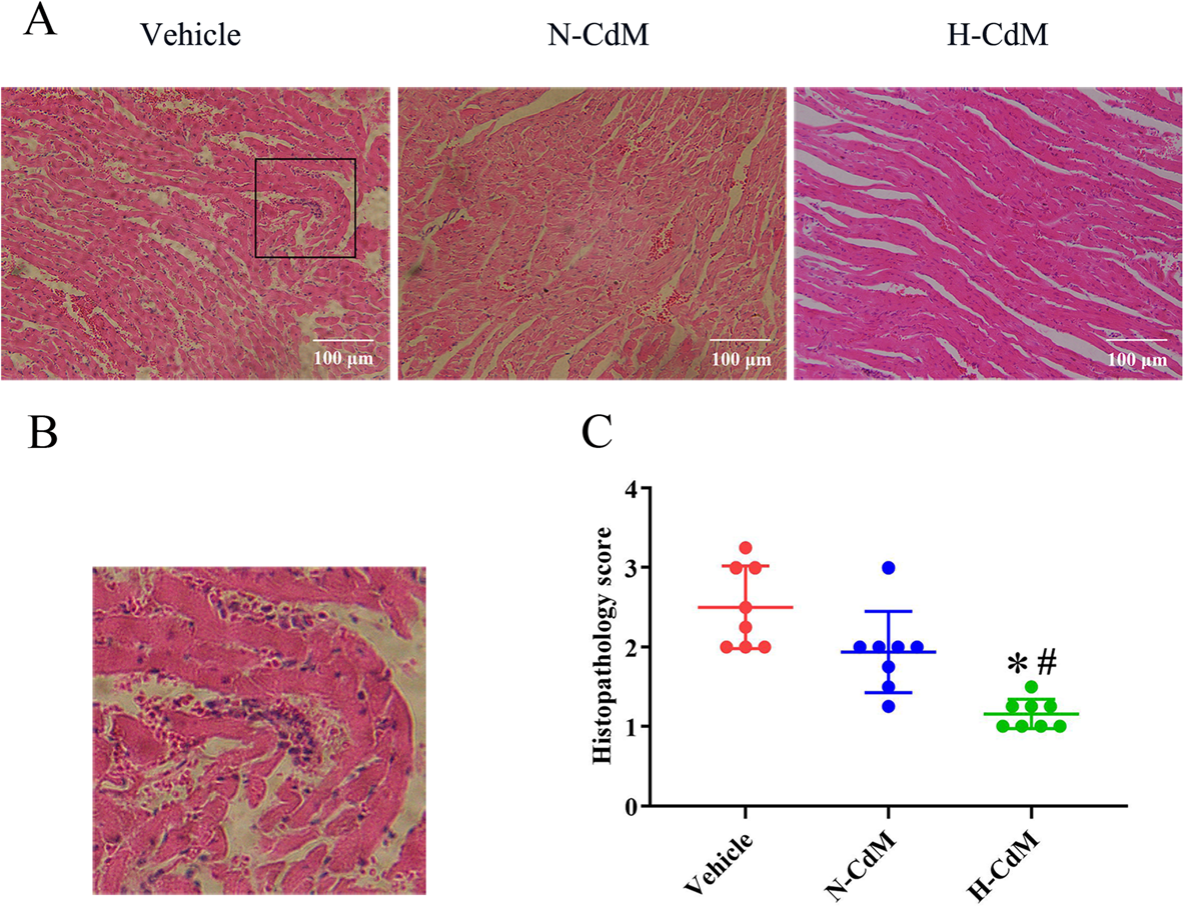
Effects of N-CdM and H-CdM on histopathology score of the donor hearts after heart transplantation. **a** Representative photomicrographs of hematoxylin and eosin (HE) staining (magnification ×200; scale bar 100 um). **b** Schematic diagram of damaged myocardium in HE staining. **c** Histopathology score of myocardial tissue for three groups. N-CdM and H-CdM indicate normoxic and hypoxic conditioned medium, respectively. Data represent mean ± standard error of the mean. One-way ANOVA/Kruskal-Wallis was applied in statistical analysis. *p < 0.05 vs. vehicle, #*p* < 0.05 vs. N-CdM

After heart transplantation, histological evaluation of hematoxylin and eosin-stained myocardial tissue demonstrated more inflammatory cell infiltration, irregularly arranged, and disordered-structure myocardial fibers in the vehicle group when compared with N-CdM and H-CdM groups (Fig. 5a, b). Hypoxic CdM-BMSCs was associated with a significantly lower histopathology score of myocardial tissue compared to vehicle and N-CdM groups (Fig. 5c). Additionally, the histopathology score showed a tendency to decrease (without reaching a significant difference) in the N-CdM group compared to the vehicle group (Fig. 5c).

#### Apoptosis in the graft

**Fig. 6:**
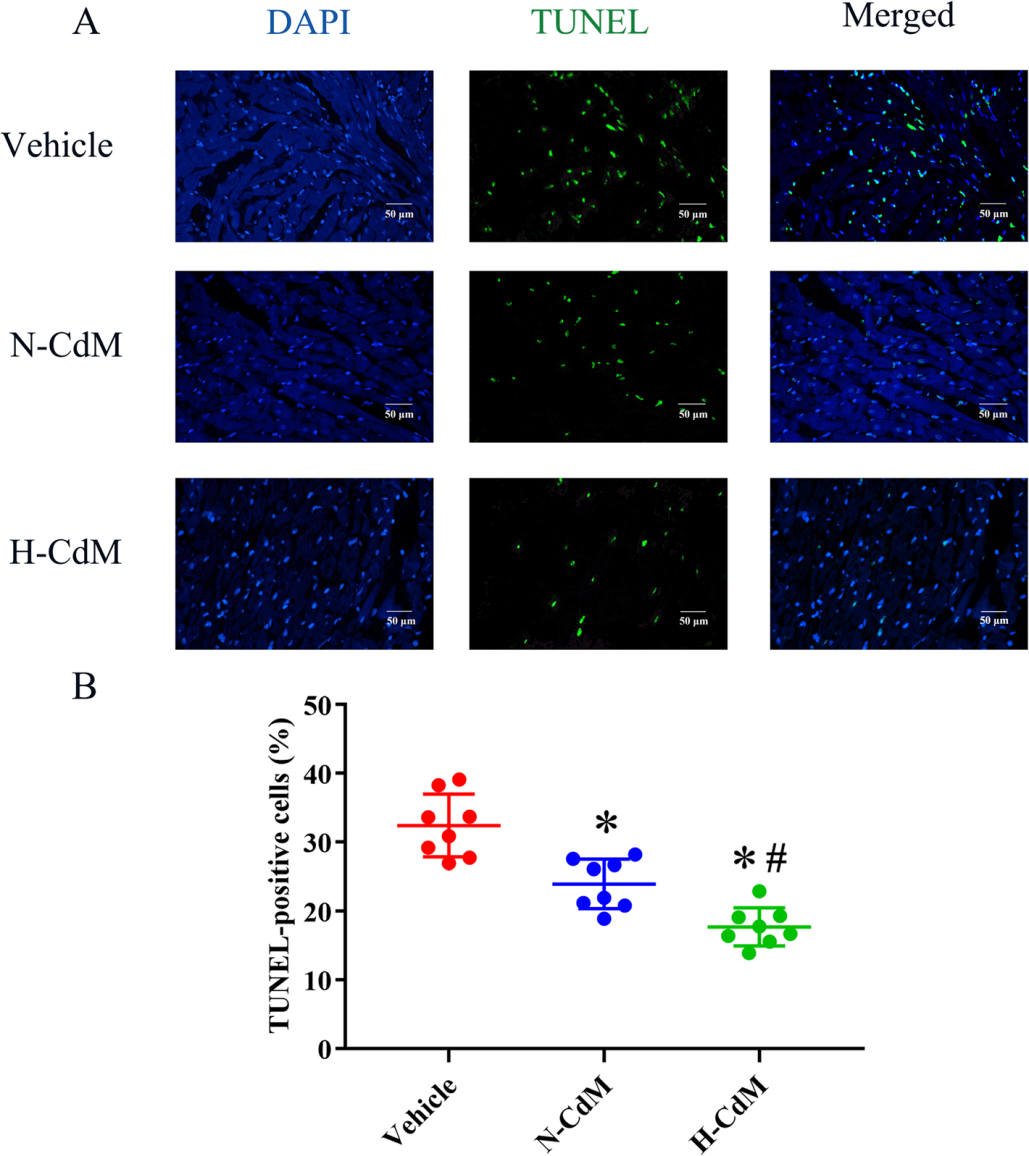
Effects of N-CdM and H-CdM on DNA strand breaks in the donor hearts after heart transplantation. **a** Representative photomicrographs of myocardial tissue stained with 4′,6-diamino-2-phenylindole (DAPI, blue), nuclei with fragmented DNA, as shown by terminal deoxynucleotidyl transferase-mediated dUTP nick end-labeling (TUNEL) staining, and merged image (magnification ×400; scale bar: 50 um). **b** Quantification of TUNEL-positive cells (as a percentage). N-CdM and H-CdM indicate normoxic and hypoxic conditioned medium, respectively. Data represent mean ± standard error of the mean. One-way ANOVA/Kruskal-Wallis was applied in statistical analysis. *p < 0.05 vs. vehicle, #*p* < 0.05 vs. N-CdM

After heart transplantation, a significantly increased number of TUNEL-positive nuclei in the graft were observed in the vehicle group compared with N-CdM and H-CdM groups (Fig. 6). Besides, treatment with hypoxic CdM-BMSCs significantly decreased DNA strand breaks in the myocardium of the grafts after heart transplantation compared with normoxic CdM-BMSCs (Fig. 6).

### Plasma level of proinflammatory cytokines and indices for myocardial injury

**Fig. 7:**
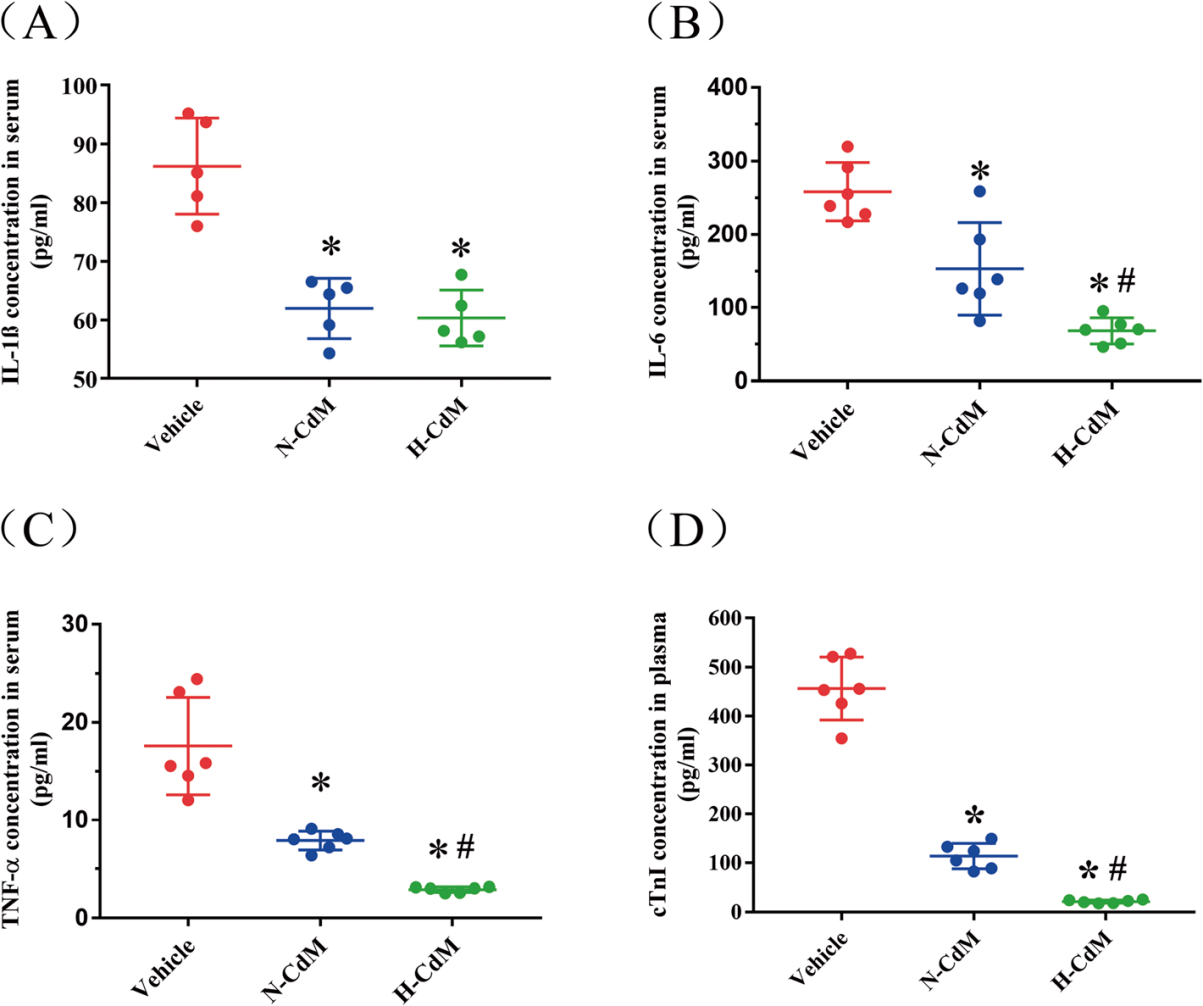
Plasma level of proinflammatory cytokines and myocardial injury indices. **a** Plasma level of Interleukin (IL)-1β. **b** Plasma level of IL-6. **c** Plasma level of tumor necrosis factor (TNF)-α. **d** Plasma level of cardiac troponin I (cTnI). N-CdM and H-CdM indicate normoxic and hypoxic conditioned medium, respectively. Data represent mean ± standard error of the mean. One-way ANOVA/Kruskal-Wallis was applied in statistical analysis. *p < 0.05 vs. vehicle, #*p* < 0.05 vs. N-CdM

The levels of proinflammatory cytokine plasma IL-1β, IL-6, and TNF-α measured at 1.5 h after reperfusion were significantly lower in the N-CdM and H-CdM groups compared with the vehicle group (Fig. 7a–c). The treatment of the donor hearts with hypoxic CdM-BMSCs significantly decreased the level of plasma IL-6 and TNF-α compared with normoxic CdM-BMSCs (Fig. 7b, c). However, no significant difference was found for plasma IL-1β between N-CdM and H-CdM groups (Fig. 7a). Additionally, circulating cTnI concentration, indicating the degree of myocardial injury, was significantly lower in the H-CdM group compared to vehicle and N-CdM groups (Fig. 7d). Moreover, the level of cTnI significantly decreased in the N-CdM group compared to the vehicle group (Fig. 7d).

### Cytokine antibody assay

**Fig. 8:**
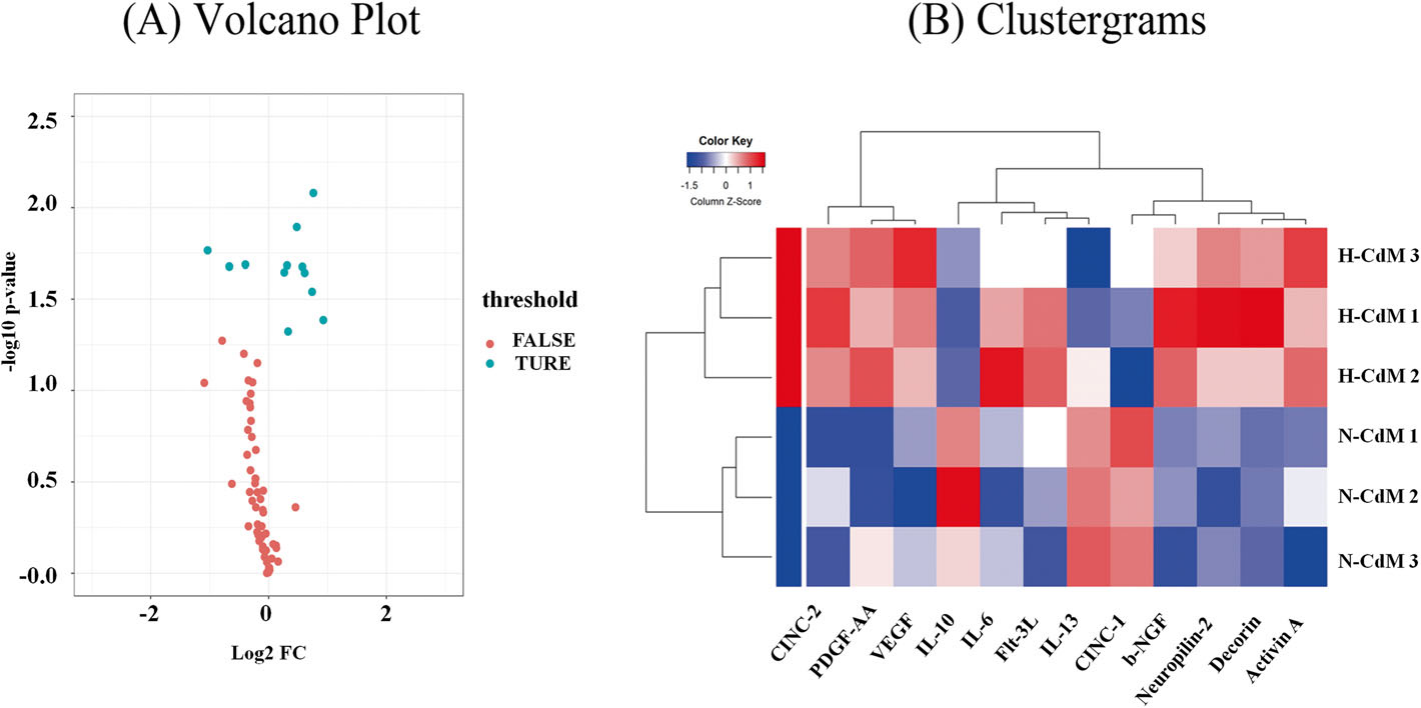
Differentially secreted cytokines levels between H-CdM and N-CdM. **a** A volcano plot shows the statistical significance of protein expression changes. The *x*-axis indicates the log_2_ of the fold changes for cytokines involved in H-CdM and N-CdM, and the *y*-axis plots − log10 of their *p* value. **b** Clustergrams create a heatmap with dendrograms to indicate all the differentially secreted cytokines between H-CdM and N-CdM. Red and blue indicate relatively high and low expression of the corresponding proteins, respectively. The *x*-axis indicates differentially expressed cytokines involved in H-CdM and N-CdM, whereas the *y*-axis indicates the rat’s number (N-CdM group includes Normoxic.1, Normoxic.2, and Normoxic.3, and H-CdM group includes Hypoxic.1, Hypoxic.2, and Hypoxic.3. *n* = 3/group). N-CdM and H-CdM indicate normoxic- and hypoxic-conditioned medium, respectively

An antibody-based protein array (67 rat cytokines) analysis was performed to investigate the component of BMSC secretome (see the supplementary material). The screening standard for differentially expressed proteins were fold changes (hypoxic/normoxic) of ≥ 1.2/1 or ≤ 1/1.2. Compared with normoxic CdM-BMSCs, there were 9 significantly increased cytokines in hypoxic CdM-BMSCs (Table 1, Fig. 8a, b). Decorin (fold change = 1.90; *p* = 0.041), cytokine-induced neutrophil chemoattractant-2 (CINC-2) (fold change = 1.69; *p* = 0.008), platelet-derived growth factor-AA (PDGF-AA) (fold change = 1.67; *p* = 0.029), neuropilin-2 (fold change = 1.53; *p* = 0.023), Activin A (fold change = 1.49; *p* = 0.021), and beta-nerve growth factor (b-NGF) (fold change = 1.39; *p* = 0.013) showed the largest differences in expression.

**Table 1:** Differentially secreted cytokines levels between hypoxic and normoxic CdM-BMSCs measured by antibody array

Protein name	EntrezID	Hypoxic CdM mean ± SD	Normoxic CdM mean ± SD	Fold change hypoxic/normoxic	***p*** value
**Decorin**	29139	10,299.06 ± 2918.47	5285.78 ± 157.00	1.901	0.041
**CINC-2**	171551	96,123.26 ± 9191.66	57,240.66 ± 10,395.19	1.692	0.008
**PDGF-AA**	25266	250,149.29 ± 24,866.75	153,260.81 ± 43,629.50	1.668	0.029
**Neuropilin-2**	81527	5556.21 ± 880.88	3613.78 ± 309.85	1.529	0.023
**Activin A**	29200	10,671.66 ± 987.40	7238.02 ± 1275.96	1.486	0.021
**b-NGF**	310738	23,323.71 ± 2472.75	16,720.57 ± 1002.78	1.391	0.013
**IL-6**	24498	661.95 ± 74.93	497.30 ± 33.82	1.327	0.026
**VEGF**	83785	246,817.39 ± 15,669.76	198,745.19 ± 16,099.43	1.243	0.021
**Flt-3 L**	103691134	915.92 ± 9.66	757.78 ± 22.63	1.209	0.0004
**IL-10**	25325	1824.10 ± 59.43	2406.64 ± 264.84	0.761	0.021
**IL-13**	116553	818.78 ± 205.59	1273.62 ± 57.60	0.630	0.021
**CINC-1**	81503	12,568.68 ± 4510.34	24,729.04 ± 2903.48	0.488	0.017

Significantly altered proteins listed first with a *p* value for the fold change. Data represent mean ± standard deviation (SD) (*n* = 3/group). *T* test/Mann-Whitney was applied in statistical analysis

*CdM* conditioned medium, *CdM-BMSCs* conditioned medium-derived from bone marrow mesenchymal stem cells

**Fig. 9:**
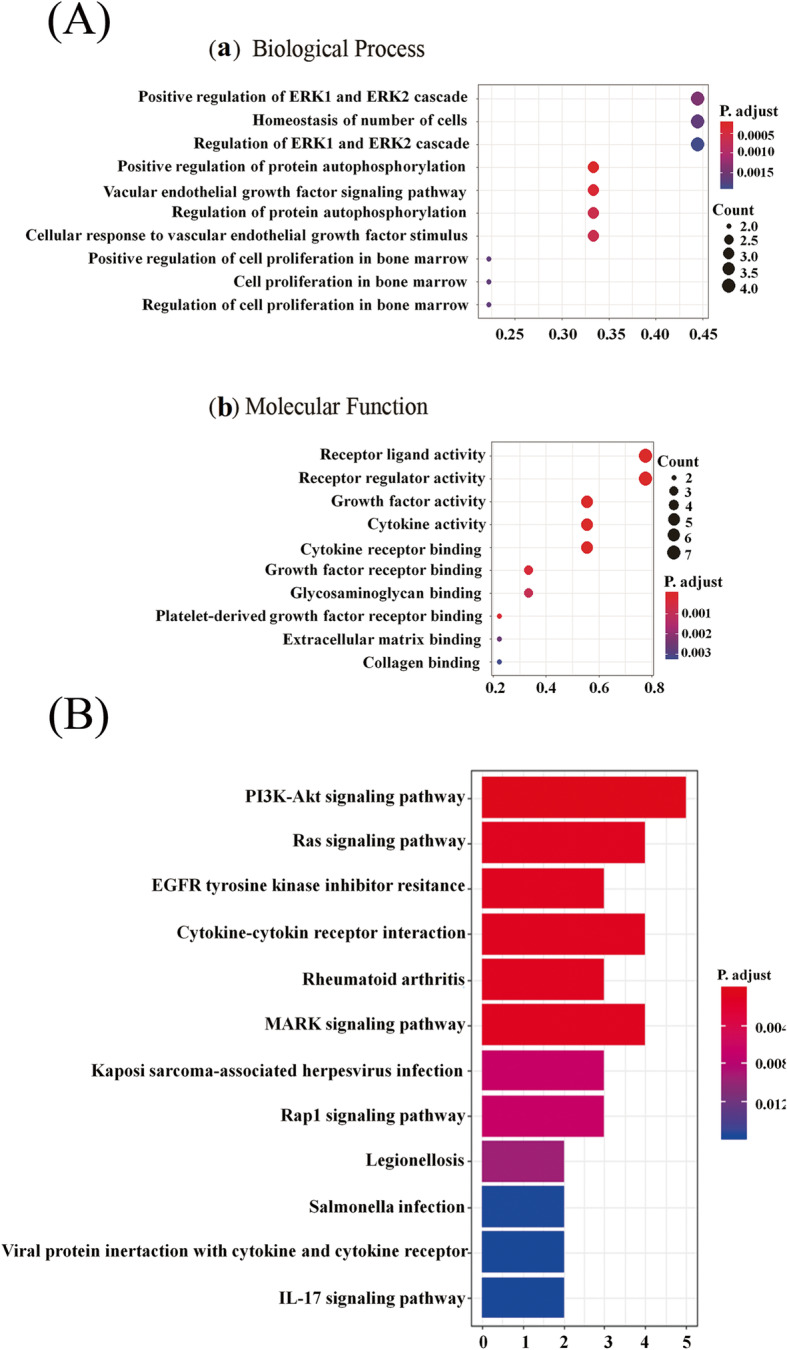
Bioinformatics analysis of the highly secreted cytokines levels between H-CdM and N-CdM. **a** The biological processes (**a**), and molecular functions (**b**) of highly expressed proteins in the H-CdM group compared with the N-CdM group based on Gene Ontology (GO) analysis. **b** Identification of signaling pathways associated with highly expressed proteins in the H-CdM group compared with the N-CdM group based on the Kyoto Encyclopedia of Genes and Genomes (KEGG) pathway enrichment analysis. The *x*-axis indicates the number of highly expressed proteins involved in the specific pathways, and the *y*-axis plots signaling pathways where the highly expressed proteins in the H-CdM group are involved. N-CdM and H-CdM indicate normoxic and hypoxic conditioned medium, respectively

GO term enrichment analysis was performed to evaluate the biological value of increased proteins in secretome derived from hypoxic-preconditioning BMSCs. As for the biological process, the upregulated proteins significantly enriched in positive regulation of ERK1 and ERK2 cascade, positive regulation of vascular endothelial growth factor signaling pathway, and positive regulation of protein autophosphorylation (Fig. 9a). With regard to molecular function, the highly expressed proteins significantly enriched in receptor ligand activity, receptor regulator activity, growth factor activity, cytokine activity, cytokine receptor binding, and growth factor receptor binding (Fig. 9a). The KEGG pathway enrichment analysis demonstrated that various signaling pathways were associated with increased proteins in hypoxic CdM-BMSCs compared to normoxic CdM-BMSCs, such as PI3K/Akt signaling pathway (adjusted *p* value = 0.00032) (Fig. 9b).

### Signaling pathways involved in the cardioprotective effects of hypoxic CdM-BMSCs

**Fig. 10:**
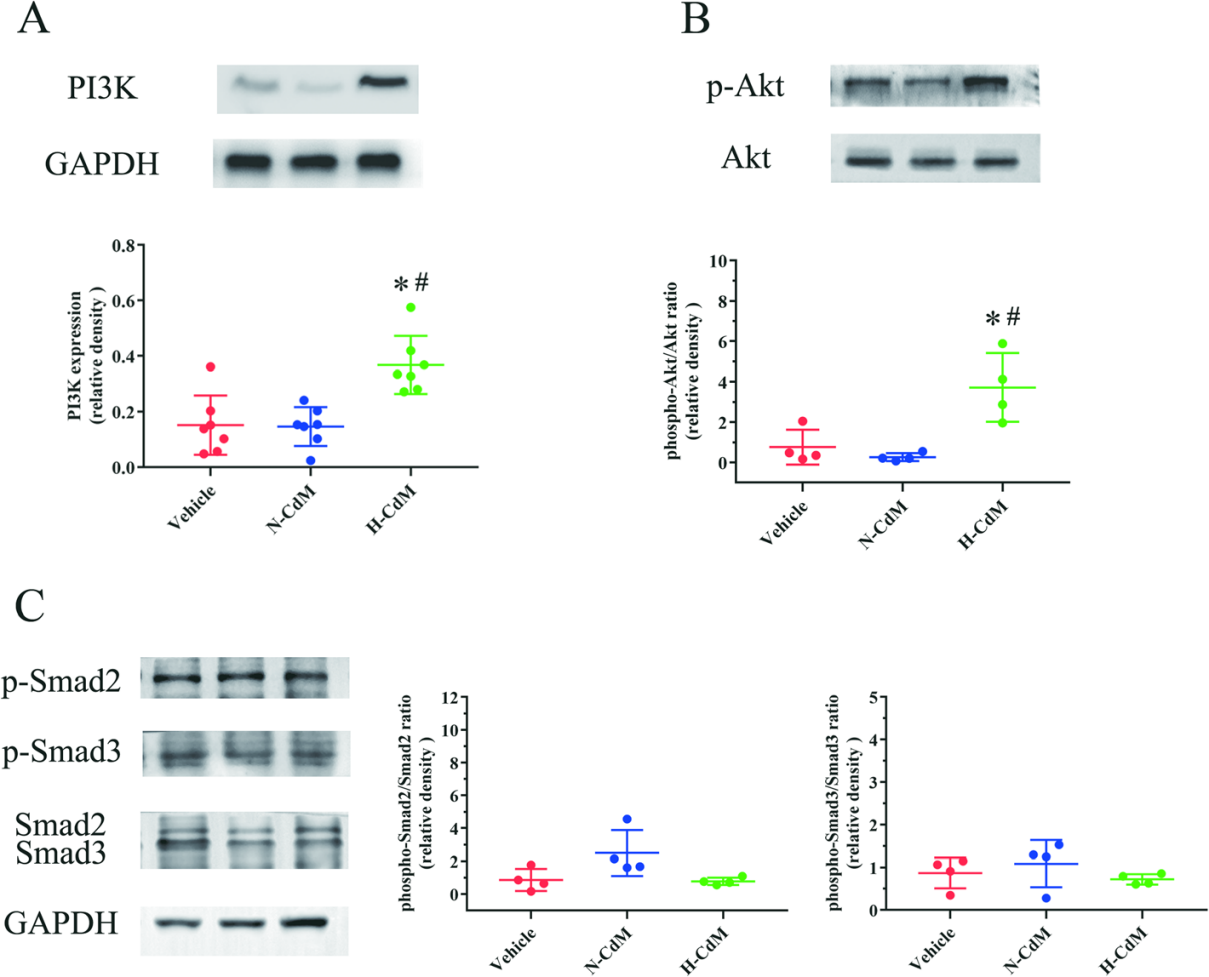
Molecular mechanisms involved in the beneficial effects of H-CdM on the donor hearts. **a** PI3K, **b** phosphorylated Akt/total Akt ratio, and **c** phosphorylated Smad2/total Smad2 and Smad3/total Smad3 ratio protein band densities in the transplanted hearts. N-CdM and H-CdM indicate normoxic and hypoxic conditioned medium, respectively. Data represent mean ± standard error of the mean. One-way ANOVA/Kruskal-Wallis was applied in statistical analysis. *p < 0.05 vs. vehicle, #*p* < 0.05 vs. N-CdM

Our KEGG analysis demonstrated that PI3K/Akt signaling might participate in the protective effect of hypoxic CdM-BMSCs against graft dysfunction associated with I/R injury in heart transplantation (Fig. 9b). Therefore, the protein expressions of PI3K, Akt, and p-Akt by Western blotting were further investigated. The results showed that hypoxic CdM-BMSCs added to the preservation solution significantly increased the protein levels of PI3K (Fig. 10a) and p-Akt/Akt ratio (Fig. 10b) in the transplanted hearts compared with the vehicle group and N-CdM groups. However, no significant difference was observed between the vehicle and N-CdM groups with regard to the protein levels of PI3K (Fig. 10a) and p-Akt/Akt ratio (Fig. 10b). Our results indicated that the beneficial effects of hypoxic CdM-BMSCs on donor heart preservation might partly result from the activation of the PI3K/Akt signaling pathway.

Since Decorin (involved in the TGF-β signaling) and Activin A (a member of TGF-β superfamily), which were involved in the Smad2/3 activation (phosphorylation) [31, 32], significantly increased in hypoxic CdM-BMSCs compared to the normoxic CdM-BMSCs in our antibody array, we further investigated the Smad2/3 activation (two downstream mediators of TGF-β signaling) in the transplanted hearts for the three groups. The results demonstrated that no significant difference was found in the phosphorylation of Smad2 and Smad3 for the donor hearts among the three groups (Fig. 10c).

## Discussion

In the present study, we investigated the role of Custodiol-supplemented with normoxic and hypoxic CdM-BMSCs as a novel preservation solution to attenuate I/R injury-induced graft dysfunction in a rat heart transplantation model. The novelty of our work is the comparison of the cardioprotective effect of normoxic and hypoxic CdM-BMSCs on the donor heart preservation. In the present study, the application of Custodiol-supplemented with normoxic CdM-BMSCs in the cold storage of the donor hearts was associated with a shorter time to return of spontaneous contraction, higher post-transplant cardiac contractility, and lower histopathological scores of the transplanted hearts. The cardioprotective effects might be associated with the inhibition of apoptosis and inflammation, as reflected by less TUNEL-positive cells and lower levels of plasma proinflammatory cytokines (interleukin-1β, interleukin-6, tumor necrosis factor-α) and cardiac troponin I in the N-CdM group compared with the vehicle group. Additionally, these beneficial cardioprotective effects are significantly enhanced after hypoxic preconditioning.

In heart transplantation, the donor hearts inevitably suffer from cold ischemia and warm reperfusion injury, which can result in PGD for transplanted hearts. I/R injury is a critical pathological process influencing graft clinical outcomes [33] due to intracellular calcium overload, the generation of ROS, inflammatory cytokines, and oxidative stress, and the absence of ATP [34]. On the other hand, apoptosis and inflammation are regarded to be involved in the pathogenesis of I/R injury, leading to tissue damage of the donor hearts. Therefore, the pre-conditioning with preservation solution has been demonstrated to be a promising method to protect the donor hearts against I/R injury-induced graft dysfunction via the inhibition of inflammation and apoptosis.

### The cardioprotective effects of normoxic CdM-BMSCs on the transplanted hearts

Recently, an increasing number of studies have demonstrated that BMSCs can secrete a vast array of paracrine factors, including cytokines, microRNA, growth factors, antioxidants, proteasomes, and exosomes [35], which are thought to be responsible for the observed cardioprotective effects for I/R injury-induced infarcted hearts in myocardial infarction [13, 36], brain-dead [23], and older [15] donor hearts in a rat heart transplantation model. In the present study, our antibody-based protein array analyses showed that the normoxic CdM-BMSCs isolated by our group contained various kinds of cytokines, including Monocyte Chemoattractant Protein-1 (MCP-1), Tissue Inhibitor of Metalloproteinase (TIMP)-1, Interleukin-10 (IL-10), Decorin, Activin A, Galectin-1, HGF, Interleukin-6 (IL-6), and VEGF, which may be involved in cardiac protection and functional improvement observed in the present study. It has been demonstrated that MCP-1 can attenuate LV dysfunction induced by global I/R injury via a reactive oxygen species-dependent but K (ATP) channel-independent pathway in the Langendorff-perfused hearts of wild-type mice [37]. Additionally, the transplantation of TIMP-1 gels into the ischemic myocardium of rats has been shown to improve cardiac function and myocardial remodeling and suppress myocardial apoptosis [38]. Furthermore, the administration of IL-10 has been suggested to improve myocardial function after acute global I/R injury and suppress inflammation via the STAT3 pathway [39]. Li et al. showed that Decorin gene therapy attenuated cardiac remodeling and dysfunction via the inhibition of Smad2/3 activation [31]. What’s more, Oshima et al. reported that the treatment of recombinant Activin A mitigated hypoxia/reoxygenation-induced apoptosis via the upregulation of Bcl-2 protein expression [40]. Besides, the treatment of mice with recombinant Galectin-1 attenuated cardiac damage by preventing cardiac inflammation in a mouse model of acute myocardial infarction [41]. Moreover, Rong et al. demonstrated that adenovirus containing human HGF gene therapy attenuated ventricular remodeling in rat hearts after myocardial infarction through reducing myocardial inflammation [42]. It also should be mentioned that IL-6 leads to a PI3K and NO-dependent protection of cardiomyocytes. This protection is associated with alterations in mitochondrial Ca2+ handling, inhibition of reperfusion-induced mitochondrial depolarization, swelling and loss of structural integrity, and suppression of cytosolic Ca2+ transients [43]. Furthermore, Yin et al. revealed that injection of VEGF carrying plasmid into myocardium restored cardiac performance and reduced infarct size and cardiomyocyte apoptosis in a rat model of myocardial infarction [44]. Last but not the least, CdM derived from human multipotent MSCs has been reported to attenuate apoptosis of human endothelial cells submitted to hypoxia due to the existence of IL-6, VEGF, and MCP-1 contained in CdM [45]. Consistent with these results and previous studies [15, 23], our study demonstrated that the “cocktail therapy” effects of a vast array of cytokines identified in normoxic CdM-BMSCs might contribute to donor heart preservation, thereby improving cardiac function, as reflected by significantly increased left ventricular EF and FS, and decreased LVIDs in echocardiography, decreasing DNA-breaks and DNA fragmentation in the donor hearts, as evidenced by TUNEL staining, and reducing the level of plasma proinflammatory cytokines (TNF-α, IL-1β, IL-6) in donor rats after heart transplantation. Therefore, our findings suggested Custodiol-supplemented with normoxic CdM-BMSCs could be introduced as a novel preservation solution for the donor hearts in the future, thereby providing a rational basis for cell-free therapy for donor heart preservation.

### The cardioprotective effects of hypoxic CdM-BMSCs on the transplanted hearts

Recent studies have shown that hypoxic preconditioning can intensify the paracrine effects of BMSCs, thereby promoting BMSCs to secrete more nutritional bioactive factors, such as VEGF, HGF, and Activin A [17, 18]. Until now, no studies have applied hypoxic CdM-BMSCs into Custodiol as a preservation solution for the cold storage of donor hearts. Therefore, we further compared the composition of the secretome, which was secreted from BMSCs into the conditioned medium under either normoxic or hypoxic conditions, to determine differences that may underlie the improved post-transplant cardiac function.

In the present study, the cardioprotective effect of the donor hearts from CdM-BMSCs could be enhanced by hypoxic preconditioning. Compared with normoxic CdM-BMSCs, the application of Custodiol-supplemented with hypoxic CdM-BMSCs in the cold storage of the donor hearts before heart transplantation was associated with a shorter time to return of spontaneous contraction, lower histopathological scores, higher post-transplant cardiac contractility, a lower rate of DNA-breaks and DNA fragmentation in the myocardium of donor hearts, and a lower level of plasma proinflammatory cytokines (IL-6, TNF-α) and indices for myocardial injury (cTnI). In line with the above findings, recent studies have shown hypoxic CdM-BMSCs can be used to treat retinal ischemia [20] and ischemic stroke [19] by exerting a more robust ant apoptosis effect. Furthermore, Xia et al. reported that hypoxia-preconditioned adipose-derived mesenchymal stem cells accelerated the repair of gastric mucosal injury through suppressing inflammation [46]. Additionally, it has been found that hypoxic CdM-BMSCs can attenuate oxygen-glucose deprivation/reoxygenation-induced injury and promote the anti-inflammatory polarization of microglia due to the beneficial effect of exosome [21].

The highly secreted soluble factors in hypoxic CdM-BMSCs may account for improved post-transplant cardiac function through the inhibition of apoptosis and inflammation. The antibody array in the present study indicated that hypoxic preconditioning resulted in an increased expression of nine cytokines. Among these identified cytokines, VEGF [44] and Activin A [40] have been demonstrated to have anti-apoptosis properties in I/R injury-induced myocardium. Additionally, Decorin secreted by human umbilical cord blood-derived mesenchymal stem cell was found to polarize inflammatory macrophages into anti-inflammatory macrophages to mitigate hyperoxic lung injury [47].

### Molecular mechanisms involved in the beneficial effects of hypoxic CdM-BMSCs

In the present study, GO term enrichment analysis showed that the biological process of increased proteins in hypoxic CdM-BMSCs included the positive regulation of ERK1/2 cascade, positive regulation of vascular endothelial growth factor signaling pathway, and positive regulation of protein autophosphorylation. Interestingly, previous studies reported that the activation of ERK1/2 cascade [48] and vascular endothelial growth factor signaling pathways [49] were associated with the attenuation of I/R injury. These results showed that the application of Custodiol-supplemented with hypoxic CdM-BMSCs for donor heart preservation protected graft against I/R injury via different signaling pathways. Further studies can be carried out to investigate the contributions of these pathways involved in the cardioprotective effect of hypoxic CdM-BMSCs. Additionally, our KEGG pathway enrichment analysis demonstrated that various signaling pathways were associated with increased proteins in hypoxic CdM-BMSCs. Intriguingly, some of these signaling pathways are involved in the mitigation of myocardial I/R injury. Among these pathways, it should be mentioned that the PI3K/Akt pathway participates in the protective mechanism of ischemia preconditioning [50]. PI3Ks are involved in the regulation of cell growth, proliferation, survival, and migration [51], whereas the phosphorylation of Akt, which is downstream of PI3K, can attenuate I/R injury [52]. In our present study, the treatment of the donor hearts with hypoxic CdM-BMSCs was associated with the increased protein levels of PI3K and p-Akt/Akt ratio compared with N-CdM and vehicle groups. Therefore, hypoxic CdM-BMSCs might exert a more robust cardioprotective effect for the donor hearts after heart transplantation partly through activating PI3K/Akt pathway. However, no significant difference was observed for the protein levels of PI3K and p-Akt/Akt ratio between the vehicle and N-CdM groups. This finding might indicate the PI3K/Akt pathway was not the primary mechanism underlying the beneficial effects of normoxic CdM-BMSCs on donor heart preservation, which was also concluded from the recent study [16].

Interestingly, no significant difference was found in the phosphorylation of Smad2 and Smad3 for the donor hearts among the three groups. Although our antibody array showed that H-CdM contained a higher level of Decorin and Activin A compared with N-CdM, Decorin [31] and Activin A [32] were found to have an opposite effect for the activation of Smad2/3 (Inhibition for Decorin, while promotion for Activin A), thereby participating in the process of cardiac fibrosis. Therefore, the phosphorylation of Smad2 and Smad3 may not be involved in the cardioprotective effect of normoxic and hypoxic CdM-BMSCs on donor heart preservation.

### Possible clinical implications and limitations

There are several limitations to our present studies. Firstly, although our rat model of heterotopic heart transplantation has been introduced as a suitable model to study global myocardial I/R injury, the LV unloading can lead to donor heart atrophy, thrombus formation in LV cavities, and faster myocardial recovery after I/R injury, which may not fully represent the real world of transplanted hearts. Secondly, the dose-response relationship between both normoxic and hypoxic CdM-BMSCs and beneficial effects for the donor heart preservation remains unclear. Thirdly, the present study was unable to identify precisely the specific cytokines or signaling pathways that account for the therapeutic effects of hypoxic CdM-BMSCs for cold storage of the donor hearts. To fully understand the precise mechanisms, future investigations can be performed by overexpressing or inhibiting some of the specific cytokines in hypoxic CdM-BMSCs. Fourthly, the impact of longer cold ischemia time for the donor hearts and the effect of CdM-BMSCs on right ventricle structure and function were not evaluated.

Despite these limitations, the current study demonstrated promising potential for the hypoxic CdM-BMSC-based therapeutic strategy against myocardial I/R injury in heart transplantation. From a clinical perspective, preconditioning heart preservation solution to mitigate myocardial I/R injury during heart transplantation can reduce the incidence of PGD for donor hearts, thereby improving short- and long-term graft function and recipient’s survival. Thus, one of the most promising and novel alternatives may be the supplementation of heart preservation solution with secretome derived from BMSCs under either normoxic or hypoxic conditions. Compared with living BMSCs, CdM-BMSCs can provide some advantages, including fewer complications for the donor hearts (carcinogenic risk, immunological, and ethical problems), convenience for storage, and transportation. However, before its successful clinical translation, some key issues should be solved, including the verification of optimal concentration and cardioprotective effect of hypoxic CdM-BMSCs on the donor hearts preservation in a big animal model, the standardization of BMSC cultivation and hypoxic preconditioning BMSCs, isolation, storage, and transportation of CdM-BMSCs in the clinical setting.

## Conclusions

Taken together, the present in vivo study reveals that the preservation of the donor hearts with cardioplegic solution supplemented with hypoxic CdM-BMSCs significantly improves post-transplant cardiac graft contractility compared with normoxic CdM-BMSCs in a rat model of heterotopic heart transplantation via the inhibition of apoptosis and inflammation. Furthermore, hypoxic-preconditioning BMSCs confers the ability to upregulate some cytokines which are associated with various potential molecular mechanisms underlying hypoxic CdM-BMSC-mediated protection against myocardial I/R injury for the donor hearts. In summary, our results indicate that the cardioplegic solution-enriched with hypoxic CdM-BMSCs can be a novel and promising preservation solution for the donor hearts.

## Supplementary Information

**Additional file 1.** : Table 1. The secreted cytokine levels between hypoxic and normoxic CdM-BMSCs measured by antibody array

## Data Availability

All data generated or analyzed during this study are included in this published article.

## Abbreviations

ATP: Adenosine triphosphate
BMSCs: Bone marrow mesenchymal stem cells
b-NGF: Beta-nerve growth factor
CO: Cardiac output
CdM: Conditioned medium
CdM-BMSCs: Conditioned medium derived from bone marrow mesenchymal stem cells
CINC-2: Cytokine-induced neutrophil chemoattractant-2
cTnI: Cardiac troponin I
DAPI: 4′,6-Diamidino-2-phenylindole
EF: Ejection fraction
FS: Fractional shortening
GO: Gene ontology
H-CdM: Hypoxic conditioned medium
HGF: Hepatocyte growth factor
HR: Heart rate
IL: Interleukin
IL-6: Interleukin-6
IL-10: Interleukin-10
I/R: Ischemia/reperfusion
KEGG: Kyoto Encyclopedia of Genes and Genomes
LVIDd: Left ventricular internal diameter at end-diastole
LVIDs: Left ventricular internal diameter at end-systole
MCP-1: Monocyte chemoattractant protein-1
N-CdM: Normoxic conditioned medium
PDGF-AA: Platelet-derived growth factor-AA
PGD: Primary graft dysfunction
PI3K: Phosphatidylinositol 3-kinase
ROS: Reactive oxygen species
SEM: Standard error of the mean
SV: Stroke volume
TIMP: Tissue inhibitor of metalloproteinase
TNF: Tumor necrosis factor
TUNEL: Terminal deoxynucleotidyl transferase dUTP nick end labeling
VEGF: Vascular endothelial growth factor
