# Supplementary material for: Donor heart preservation with hypoxic-conditioned medium-derived from bone marrow mesenchymal stem cells improves cardiac function in a heart transplantation model
Source: Stem Cell Res Ther. 2021 Jan 13;12:56. doi: 10.1186/s13287-020-02114-7 (PMC7805188; doi:10.1186/s13287-020-02114-7)
Supplement: Supplementary file 1 — Additional file 1. : Table 1. The secreted cytokine levels between hypoxic and normoxic CdM-BMSCs measured by antibody array [file 13287_2020_2114_MOESM1_ESM.doc]

**TABLE 1 The secreted cytokines levels between Hypoxic and Normoxic CdM-BMSCs measured by antibody array**

| **Protein name** | **EntrezID** | **Hypoxic CdM**  **Mean ± SD** | **Normoxic CdM**  **mean ± SD** | **Fold change**  **hypoxic/normoxic** | **p value** |
| --- | --- | --- | --- | --- | --- |
| **Decorin** | 29139 | 10,299.06±2,918.47 | 5,285.78±157.00 | 1.901 | 0.041 |
| **CINC-2** | 171551 | 96,123.26±9,191.66 | 57,240.66±10,395.19 | 1.692 | 0.008 |
| **PDGF-AA** | 25266 | 250,149.29±24,866.75 | 153,260.81±43,629.50 | 1.668 | 0.029 |
| **Neuropilin-2** | 81527 | 5,556.21±880.88 | 3,613.78±309.85 | 1.529 | 0.023 |
| **Activin A** | 29200 | 10,671.66±987.40 | 7,238.02±1,275.96 | 1.486 | 0.021 |
| **b-NGF** | 310738 | 23,323.71±2,472.75 | 16,720.57±1,002.78 | 1.391 | 0.013 |
| **IL-6** | 24498 | 661.95±74.93 | 497.30±33.82 | 1.327 | 0.026 |
| **VEGF** | 83785 | 246,817.39±15,669.76 | 198,745.19±16,099.43 | 1.243 | 0.021 |
| **Flt-3L** | 103691134 | 915.92±9.66 | 757.78±22.63 | 1.209 | 0.0004 |
| **IL-10** | 25325 | 1,824.10±59.43 | 2,406.64±264.84 | 0.761 | 0.021 |
| **IL-13** | 116553 | 818.78±205.59 | 1,273.62±57.60 | 0.630 | 0.021 |
| **CINC-1** | 81503 | 12,568.68±4,510.34 | 24,729.04±2,903.48 | 0.488 | 0.017 |
| **Gas 1** | 683470 | 6,243.81±3,046.25 | 4,124.29±470.94 | 1.387 | 0.300 |
| **TIMP-2** | 29543 | 371.83±133.04 | 366.15±124.86 | 1.013 | 0.960 |
| **MCP-1** | 24770 | 221,999.23±109,781.95 | 204,467.62±122,193.84 | 1.118 | 0.862 |
| **Galectin-3** | 83781 | 1,009.80±185.95 | 938.11±274.71 | 1.095 | 0.727 |
| **LIX** | 60665 | 2,114.35±347.94 | 1,934.61±283.31 | 1.091 | 0.526 |
| **Galectin-1** | 56646 | 20,325.03±1,945.30 | 19,365.81±3,390.65 | 1.057 | 0.693 |
| **TIMP-1** | 116510 | 288,660.57±43,707.23 | 279,543.74±53,416.56 | 1.038 | 0.830 |
| **MIP-1a** | 25542 | 455.49±101.46 | 492.22±181.63 | 0.957 | 0.775 |
| **Notch-2** | 29492 | 9,445.30±4,466.48 | 9,150.09±3,182.28 | 1.002 | 0.930 |
| **HGF** | 24446 | 1,017.45±114.11 | 1,011.19±53.67 | 1.003 | 0.936 |
| **Fractalkine** | 89808 | 15,474.18±5,917.66 | 15,273.07±4,710.26 | 0.996 | 0.965 |
| **IL-1 R6** | 171106 | 1,190.58±304.09 | 1,190.28±47.25 | 0.980 | 0.999 |
| **Erythropoietin** | 24335 | 431.13±62.54 | 439.72±57.19 | 0.979 | 0.869 |
| **Adiponectin** | 246253 | 835.32±68.78 | 865.43±137.17 | 0.971 | 0.751 |
| **B7-2** | 56822 | 1,129.88±108.43 | 1,165.71±25.70 | 0.967 | 0.607 |
| **RANTES** | 81780 | 1,076.89±185.52 | 1,229.61±497.42 | 0.919 | 0.644 |
| **EphA5** | 79208 | 1,029.24±52.12 | 1,096.62±98.00 | 0.940 | 0.352 |
| **TWEAK R** | 302965 | 7,045.39±739.24 | 7,485.19±585.16 | 0.940 | 0.464 |
| **IL-1b** | 24494 | 1,256.14±285.28 | 1,329.46±219.34 | 0.937 | 0.742 |
| **gp130** | 25205 | 1,271.46±257.44 | 1,358.78±284.42 | 0.935 | 0.713 |
| **IL-1 ra** | 60582 | 535.95±31.46 | 578.12±132.48 | 0.942 | 0.620 |
| **TCK-1** | 246358 | 1,998.06±247.57 | 2,132.01±125.34 | 0.933 | 0.450 |
| **Prolactin** | 24683 | 556.13±8.51 | 587.90±84.55 | 0.952 | 0.553 |
| **IL-7** | 25647 | 586.71±128.06 | 639.43±125.89 | 0.916 | 0.638 |
| **ICAM-1** | 25464 | 4,475.13±560.14 | 4,651.26±85.38 | 0.957 | 0.619 |
| **IL-2** | 116562 | 2,313.65±302.90 | 2,540.73±276.12 | 0.909 | 0.392 |
| **IL-1a** | 24493 | 1,617.77±495.00 | 1,780.12±347.62 | 0.895 | 0.666 |
| **CNTF** | 25707 | 1,564.30±472.04 | 1,718.45±141.02 | 0.885 | 0.617 |
| **TREM-1** | 301229 | 1,044.69±246.62 | 1,189.53±280.78 | 0.878 | 0.539 |
| **SCF** | 60427 | 442.55±82.59 | 501.20±53.26 | 0.876 | 0.360 |
| **IL-3** | 24495 | 780.98±33.63 | 892.91±71.73 | 0.876 | 0.071 |
| **P-Cadherin** | 116777 | 459.61±143.36 | 515.71±88.02 | 0.873 | 0.594 |
| **JAM-A** | 116479 | 426.14±96.34 | 492.57±91.21 | 0.861 | 0.435 |
| **B7-1** | 25408 | 654.37±95.71 | 757.78±72.85 | 0.860 | 0.211 |
| **CTACK** | 362505 | 1,219.78±251.59 | 1,404.79±101.77 | 0.857 | 0.303 |
| **IL-17F** | 301291 | 420.39±45.63 | 504.55±28.74 | 0.831 | 0.054 |
| **Notch-1** | 25496 | 1,159.72±216.60 | 1,354.53±204.98 | 0.852 | 0.321 |
| **GFR alpha-1** | 25454 | 666.31±80.45 | 802.75±69.58 | 0.828 | 0.090 |
| **Neuropilin-1** | 246331 | 499.48±154.80 | 585.78±36.97 | 0.826 | 0.401 |
| **CD48** | 245962 | 560.17±98.38 | 680.02±81.69 | 0.820 | 0.180 |
| **IL-22** | 500836 | 425.09±71.95 | 519.67±55.98 | 0.814 | 0.147 |
| **IL-2 Ra** | 25704 | 417.92±63.17 | 514.12±48.37 | 0.809 | 0.104 |
| **L-Selectin** | 29259 | 1,545.69±405.94 | 1,878.73±201.93 | 0.808 | 0.272 |
| **FGF-BP** | 64535 | 332.28±59.20 | 409.15±34.46 | 0.806 | 0.124 |
| **IFNg** | 25712 | 764.65±128.94 | 948.15±94.22 | 0.802 | 0.117 |
| **Nope** | 363081 | 1,738.54±459.88 | 2,156.56±526.19 | 0.800 | 0.359 |
| **Eotaxin** | 29397 | 347.66±14.23 | 365.27±41.13 | 0.955 | 0.522 |
| **TNFa** | 24835 | 1,033.02±829.09 | 1,912.47±452.29 | 0.368 | 0.182 |
| **IL-4** | 287287 | 1,731.31±347.08 | 2,192.12±316.60 | 0.785 | 0.165 |
| **TIM-1** | 286934 | 243.93±52.12 | 314.99±68.06 | 0.776 | 0.224 |
| **Prolactin R** | 24684 | 744.40±147.91 | 958.16±109.11 | 0.770 | 0.114 |
| **4-1BB** | 500590 | 554.98±111.90 | 733.64±46.40 | 0.747 | 0.063 |
| **GM-CSF** | 116630 | 982.65±477.11 | 1,390.89±124.94 | 0.660 | 0.225 |
| **RAGE** | 81722 | 1,073.01±304.32 | 1,492.90±60.49 | 0.700 | 0.079 |
| **CINC-3** | 114105 | 17,073.14±4,595.66 | 25,512.04±11,613.59 | 0.708 | 0.307 |

Significantly altered proteins listed first with a p-value for the fold change. CdM, conditioned medium; CdM-BMSCs: conditioned medium derived from bone marrow mesenchymal stem cells. Data represent mean ± standard deviation (SD) (n = 3/group). T-test/Mann-Whitney was applied in statistical analysis.
